# Determination of ketamine using melamine-modified gold nanoparticles

**DOI:** 10.55730/1300-0527.3593

**Published:** 2023-06-07

**Authors:** Güler GÜNEŞ, Ziya CAN, Ayşem ARDA, Mustafa Reşat APAK

**Affiliations:** 1 TEBIP High Performers Program, Board of Higher Education of Turkiye, Istanbul University-Cerrapaşa, İstanbul, Turkiye; 2 Department of Chemistry, Faculty of Engineering, İstanbul University, İstanbul, Turkiye; 3 Turkish Academy of Sciences (TUBA), Ankara, Turkiye

**Keywords:** Ketamine determination, gold nanoparticles, melamine, colorimetric sensor, drug testing

## Abstract

Ketamine is used in medicine because of its anaesthetic and antidepressant effects at low doses. Unfortunately, due to its narcotic effect when used at high doses, its abuse among young people is increasing. It is also one of the most common drugs used in rape. Therefore, there is a need for fast and inexpensive tests that can be performed on-site. With the advancement of nanotechnology, nanoparticle-based approaches have found their place in selective analyses as in many fields. In the developed method, firstly gold nanoparticles were modified with melamine (AuNPs@Mel). Under optimized conditions, hydrogen bonds formed between ketamine and AuNPs@Mel cause the red colour of AuNPs@Mel to shift to blue-purple (i.e. aggregation-induced surface plasmon absorption shift). The association between absorbance and concentration produced a calibration line (curve) having a linearity correlation coefficient of 0.9981 for ketamine concentrations ranging from 4.76 to 47.6 mg L^−1^. The detection limit of the proposed method was 1.5 mg L^−1^ and the RSD (relative standard deviation) values of concentrations were changed ranging from 5.2% to 8.2%. The intra-assay and inter-assay measurements using the suggested method resulted in coefficients of variation (CVs) of 5.7% and 8.5%, respectively. Scan transmission electron microscopy (STEM), UV-vis spectrophotometry and FTIR spectroscopy were used to characterize the synthesized and modified AuNPs. Additionally, the procedure was successfully carried out with some interference materials and a real sample of fetal bovine serum. Lastly, using the Student t-test and F tests, the suggested technique was compared to and confirmed against an LC-MS/MS procedure previously published.

## 1. Introduction

Ketamine hydrochloride, also known by its IUPAC name, 2-(2-chlorophenyl)-2-(methylamino)cyclohexanone hydrochloride, is a fast-acting anaesthetic finding use in animal and human surgical operations under a secondary name of “ketalar” [1]. Currently, ketamine is not widely used for therapeutic purposes in humans, but it is used in traumas or emergency operations and is also used in veterinary medicine [2]. It can be administered orally, intramuscularly or by inhalation [3]. It is a substance that causes antidepressant effect when used at low doses and causes reduced attention, impaired learning ability, or hallucinations. At high elevated doses, it may give rise to respiratory problems, increased blood pressure, and sometimes even death [4]. According to autopsy studies conducted on people who died of ketamine overdose, the drug was found in blood, urine, brain, spleen, liver and kidney tissues [2].

Recently, the illicit use of ketamine has been increasing considerably, especially among young people [5]. Ketamine is addictive similar to cocaine [6], and causes not only psychological but also social problems. Fatal cases of ketamine poisoning have also been described in the literature [7]. Although the development of psychological dependence and tolerance is frequently observed, withdrawal symptoms rarely occur [8]. Therefore, it has become important to investigate this substance criminally. The rapid, simple, and inexpensive determination of ketamine is very important for recognizing the use of this substance.

When the literature for ketamine determination is examined, chromatographic analyses combined with different detector types come to the fore. While a mass spectrometry (MS) detector is commonly used in combination with gas chromatography [9–11], a flame ionisation detector has also been used [12]. With liquid chromatography (LC), UV [13,14], MS [15,16], and MS/MS [17,18] detectors were used. Although successful results are obtained at low detection limits with these combined techniques, the costs of the devices used are quite high, and experienced analysts are needed for their use. In addition, derivatization steps are required for compounds with poor vapor pressure behaviour in gas chromatography [19]. In addition, spectrophotometric methods based on charge-transfer complexation [20,21], fluorometric [22,23], and voltammetric methods [24, 25] were also used to analyse ketamine.

Nanotechnology is an advanced technology related to the synthesis and processing of nanoparticles (generally within the 1–100 nm range) and their applications. Due to their superior optical properties such as interparticle distance, shape, and size, AuNPs have become an essential component of colorimetric approaches, which specifically allow on-site determinations even with the naked eye. Surface plasmon resonance (SPR) absorption band density may show variations depending on AuNPs morphology (size, shape, etc.) [26,27]. As a result, alternative colorimetric sensing strategies were designed in accordance with physicochemical phenomena such as aggregation and antiaggregation. The inter-particle distance variations of AuNPs due to electron donor-acceptor, hydrogen-bonding, and electrostatic interactions with the target analyte constitute the foundation of aggregation-based colorimetric systems design [28]. Despite the fact that aggregation-based systems may not seem selective, analyses can be successfully made selective by coating gold nanoparticle surfaces with a modifier specific to the substance to be analysed so that particular physicochemical interactions prevail between the modified nanoparticles and the analyte. This strategy has proven to be a useful vantage point for the selective determination of numerous compounds. With the increasing analytical applications of nanomaterials, colorimetric determination of ketamine was carried out with an anionic cobalt(II) complex attached to nano-SiO_2_ [5]. However, since ketamine has a pK_a_ of 7.5, its cationic (ammonium) form may electrostatically interact with the nanosilica-modifier tetracyanocobaltate(II) anionic complex to produce an ion pair, and other similar cationic amines may interfere. On the other hand, spectrophotometric ketamine detection utilizing unlabelled or modified AuNPs based on the aggregation/disaggregation mechanism is almost nonexistent in the literature.

In the current study, we suggest an AuNPs@Mel based ketamine colorimetric detection method that is quick, easy, and reasonably selective. Melamine as a nitrogen-rich compound contains three outer amino substituents mounted on a 1,3,5-triazine skeleton, thus it can displace the weakly-held citrate groups situated on initially prepared AuNPs by stronger coordination bonds. While melamine at medium-to-high concentrations may cause agglomeration/aggregation of AuNPs, it may functionalize AuNPs in solution at low concentrations to produce an effective probe for target compounds by displaying charge-transfer and hydrogen bonding/supramolecular complexation capabilities toward those analytes [29]. This study’s underlying assumption is that melamine functionalized on nanoparticles interacts with ketamine through intermolecular hydrogen bonds, causing AuNPs@Mel to aggregate accompanying a bathochromic shift in the localized plasmon absorption band. The SPR absorption density at around 520 nm declined as ketamine concentrations increased, while a new absorption band emerged at about 650 nm. By correlating the ratio of the absorbance at 650 nm to the absorbance at 520 nm (referred to as “corrected absorbance”) to ketamine concentration, the analytical evaluation was carried out. Additionally, the method was carried out to some ions, possible camouflage materials, and a real sample composed of fetal bovine serum: FBS. Lastly, the proposed method was compared to a reference LC-MS/MS assay.

## 2. Materials and methods

### 2.1. Instrumentation and chemicals

Absorbance data were collected using Hellma GmbH & Co. KG (Müllheim, Germany) quartz cells with a Shimadzu UV-1900i spectrophotometer (Kyoto, Japan). An FEI Quanta 450 FEG scanning electron microscope (Oregon, USA) was used to analyse nanoparticles using scanning transmission electron microscopy (STEM). With a Tetra Jasco 6600 FT-IR spectrometer (Easton, USA), FTIR spectra were collected. While the baseline of FTIR spectra for melamine and AuNPs@Mel were taken against water, the baseline was taken against air for pure ketamine and AuNPs@Mel in the presence of ketamine. For validation of the developed method, a published LC-MS/MS method was performed with Shimadzu-8040 (Kyoto, Japan). LC analysis was performed on a Restek Ultra-AQ C18 column (Pennsylvania, USA) (100 × 2.1 mm, 3 μm). Hettich Universal 320 centrifuge (Tuttlingen, Germany) and IKA (Staufen, Germany) heater with magnetic stirring were used for both synthesizing and modifying the AuNPs. A Mettler Toledo Seven Compact S220 (Columbus, USA) pH meter was used to monitor pH levels.

All chemicals used in the experiments had an analytical reagent level purity unless stated otherwise. The ketamine hydrochloride standard was obtained from Cerilliant (Texas, USA). Trivalent gold chloride solution (99.99% trace metal basis, having a weight percentage of 30% in dilute HCl in the form of HAuCl_4_) and melamine used in synthesizing and modifying the AuNPs were bought from Sigma-Aldrich (Steinheim, Germany). FBS was supplied by Sigma-Aldrich (Steinheim, Germany), and the remaining reagents were from Merck (Darmstadt, Germany) and Sigma-Aldrich (Steinheim, Germany).

### 2.2. Preparation of solutions

Ketamine hydrochloride solutions were diluted to appropriate concentrations from the standard in ultrapure water. Melamine solution (1.0 × 10^−5^ mol L^−1^), trisodium citrate (1%, w:v), pH 4.5 acetate buffer solution (having a CH_3_COONa/CH_3_COOH total concentration of 0.1 mol L^−1^) were prepared in water (ultrapure).

Commercially available FBS was prepared by precipitation of bovine serum protein as previously described elsewhere [30]. For bovine serum protein precipitation, the following procedure was performed 5 times on the supernate until precipitation of proteins ceased: 2 mL EtOH was mixed with 3.33 mL FBS, and the final solution was subjected to centrifugation for a period of 10 min at 10,000 rpm. The obtained supernate was made up to 100 mL with ultrapure water.

The stock solutions of K^+^, Ca^2+^, Cl^−^, NO_3_^−^, and SO_4_^2−^ ions were prepared in ultrapure water. Acetylsalicylic acid (aspirin) and paracetamol-based painkillers that can be used to camouflage ketamine were prepared in methanol and filtered from a glass fiber/poly (ethylene terephthalate) microfilter. Solutions of ions and camouflage compounds were diluted from stock solutions at 500 mg L^−1^ concentration.

### 2.3. Synthesis and modification of AuNPs

The synthesis of AuNPs was made according to the conventional method of Turkevich [31]. Firstly, 50 mL of 0.01% HAuCl_4_ solution at pH 4 was brought to boiling, followed by the addition of 2 mL of 1% Na_3_Cit: trisodium citrate. Boiling was continued until the solution turned wine red, followed by the turning-off of the heater. The solution was cooled to ambient temperature. The synthesized gold nanoparticle solution was separated into 6 tubes with 8 mL in each tube and centrifuged at 10000 rpm for 40 min. The supernatants were removed to eliminate excess citrate. Finally, for dispersing the remainder part, 200 μL of water was added to it and collected all in 25 mL.

For modification, 25 mL of the synthesised AuNPs were transferred to an Erlenmeyer flask and mixed at 300 rpm for 10 min. Then, 3 mL of 1.0 × 10^−5^ mol L^−1^ solution of melamine was added and the mixing was continued for 30 minutes.

### 2.4. Proposed method for ketamine determination

The developed method for ketamine determination is summarized as follows:

500 μL of melamine modified gold nanoparticles + 50 μL of acetate buffer + 500 μL ketamine solution (water for reference solution)

At the end of 15 min, absorbance measurements were carried out, and the 520 nm and 650 nm absorbances were proportionated.

### 2.5. Preparation of fetal bovine serum-containing solutions as real samples for analysis

For ketamine analysis in fetal bovine serum (FBS) prepared by precipitation of protein was diluted 30-fold and used for dilution of ketamine samples. The suggested method was applied to the prepared ketamine solutions in the range of 10–100 mg L^−1^. Finally, the calibration curve for standard addition was obtained by adding ketamine at a concentration of 10 mg L^−1^ to the normal calibration curve. The calibration curves obtained by using pure standards and by application of the method of standard additions were plotted between ketamine concentration and corrected absorbance.

### 2.6. Investigation of effects of ions and camouflage materials

Ketamine (10 mg L^−1^, initial concentration) was analyzed in the presence of a 1-fold concentration of some ions (K^+^, Ca^2+^, Cl^−^, NO_3_^−^, and SO_4_^2−^) and possible camouflage materials (acetylsalicylic acid (aspirin) and paracetamol-based painkiller drugs). The selectivity of the proposed method was investigated and the recovery values of ketamine were calculated.

### 2.7. Evaluation of spectrophotometric data

In the developed method, absorbance calculations were performed according to the ratiometric method. Initially, the modified sensor had an absorbance at 520 nm. When ketamine determination was employed, the colour shifted to blue-violet due to aggregation of AuNPs and a new peak appeared in the 600700 nm range. Absorbance measurements of the reference solution and all ketamine-containing solutions were made against water. Absorbances at 520 and 650 nm were noted and corrected absorbances were found with respect to the equation below:


$$\text{Corrected Absorbance: }{\text{Abs}}_{\left(650/520\right)}={\left({\text{A}}_{650}/{\text{A}}_{520}\right)}_{\text{Ketamine}}-{\left({\text{A}}_{650}/{\text{A}}_{520}\right)}_{\text{Ref}}$$

### 2.8. Determination of ketamine by LC/MS-MS for statistical analysis

To validate the developed colorimetric ketamine sensing method, a literature method based on LC-MS/MS was performed [32]. To show the linearity of the LC-MS/MS method, diluted working solutions with a concentration range of 1–30 μg L^−1^ were prepared from the stock solution of ketamine. LC was carried out using a Restek Ultra-AQ C18 column (100 × 2.1 mm ID, 3 μm particle size) which was kept at 45 °C temperature. The gradient program was carried out the same as in the literature [32]. The positive ion mode electrospray ionisation method was used for LC-MS/MS analysis. The precursor ion and product ion were m/z 238.1 and 124.8 for ketamine, respectively (collision energy: 29 V). Student’s t- and F-tests were used to statistically compare the outcomes of spectrophotometry and LC-MS/MS.

## 3. Results and discussion

### 3.1. Synthesis and characterization of AuNPs

**Figure 1:**
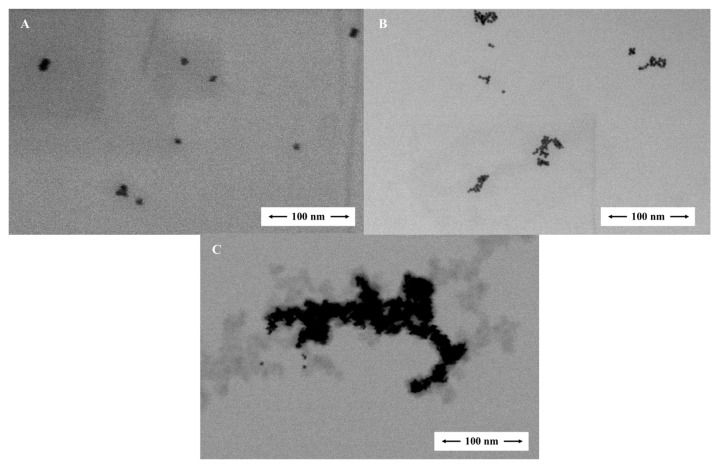
STEM images of AuNPs (A), AuNPs@Mel (B), and AuNPs@Mel in the presence of 100 mg L^−1^ ketamine (C).

**Figure 2:**
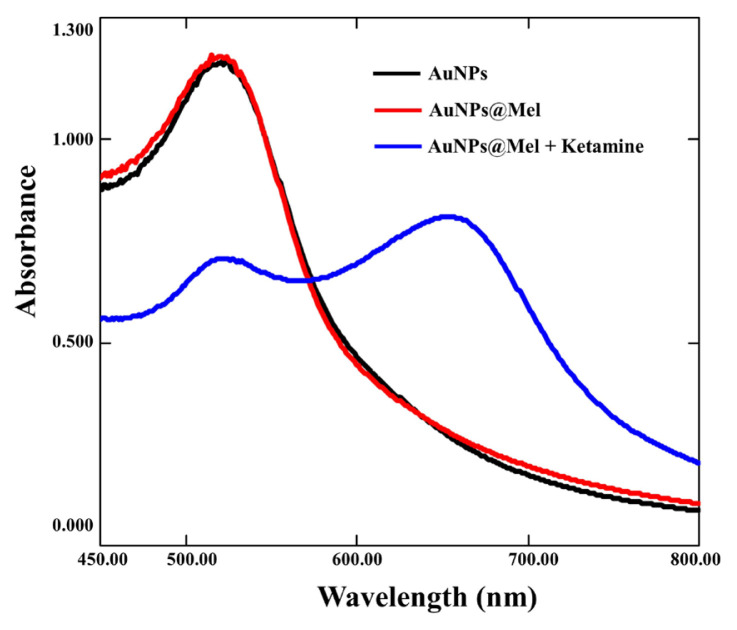
Absorbance spectrum of AuNPs (black line), AuNPs@Mel (red line), and AuNPs@Mel in the presence of 100 mg L^−1^ ketamine (blue line).

Firstly, gold nanoparticles were synthesized with respect to the classical Turkevich procedure [31]. In this method, trisodium citrate (Na_3_Cit) is used as an agent for reducing tri-valent gold to zero-valent gold, and at the same time, citrate stabilizes the formed AuNPs’ surface. As known from the literature, monodisperse AuNPs yield, particle size and synthesis reproducibility are highly pH-dependent due to the possibility of hydrolysis of citrate and chloroaurate species [33–35]. Therefore, the pH of HAuCl_4_ solution was adjusted to 4 with NaOH in the synthesis step. UV-Vis spectra were taken for the reproducibility of the synthesized nanoparticles and the plasmon absorbance at 520 nm was evaluated. As a result, reproducible values were obtained for intra-day and inter-day measurements. For modification with melamine, the synthesized gold nanoparticles were centrifuged to remove excessive citrate in the medium and were subsequently modified with melamine as described in Section 2.3. Finally, the method was applied as detailed in Section 2.4. STEM images of the synthesised, modified, and applied nanoparticles are shown in Figure 1 and their UV-Vis spectra are shown in Figure 2. According to STEM measurements, there was no remarkable difference between the mean nanoparticle diameters of original AuNPs (Figure 1 (A)) and modified AuNPs@Mel (Figure 1 (B)) (10–17 nm). However, the aggregation of AuNPs@Mel via hydrogen-bonding interaction with the addition of ketamine resulted in an increase in mean nanoparticles diameter up to 70–80 nm (Figure 1 (C)). In addition, as can be seen from the spectra in Figure 2, there was no significant change in the spectra of the modified gold nanoparticles compared to that of the unmodified ones, while a significant peak formation was observed at the wavelength of 650 nm by adding ketamine to the medium. Naturally, the choice of melamine concentration was critical at this stage because excessive concentrations may cause self-aggregation of AuNPs@Mel without analyte [29].

**Figure 3:**
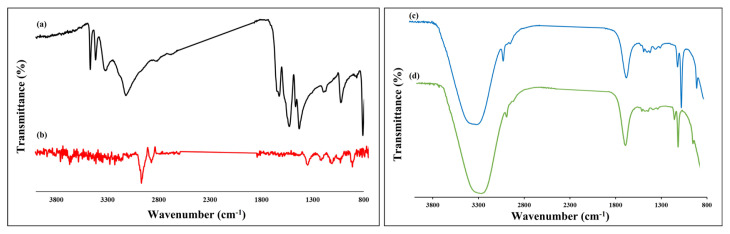
FTIR spectra of a) melamine, b) AuNPs@Mel (b), c) ketamine, and d) AuNPs@Mel in presence of ketamine.

The FTIR spectra seen in Figure 3 reveal that AuNPs are successfully modified with melamine and there is hydrogen bonding between ketamine and melamine-modified gold nanoparticles. The FTIR spectra of pure melamine (a), AuNPs@Mel (b), pure ketamine (c), and AuNPs@Mel interacted ketamine (d) were recorded. The characteristic peaks of -NH and -NH_2_ at 3000–3500 cm^−1^ in pure melamine were shifted in the AuNPs@Mel FTIR spectrum. This showed that -NH_2_ group of melamine was interlinked with AuNPs via Au-N bonds [36,37]. On the other hand, FTIR spectra of pure ketamine showed characteristic secondary amine stretching peaks at 2852–2980 cm^−1^, C=O stretching band at 1642, stretching vibrations of the C–N at 1043–1488 cm^−1^, and Cl group at 876 cm^−1^ peaks (curve c). The C-N and N-H stretching peak intensities of ketamine significantly decreased because ketamine interacted with AuNPs@Mel (curve d), as shown in Figure 3. Moreover, a decrease in the Cl peak intensity at 876 cm^−1^ was observed [37–40]. These modifications in the spectrum of AuNPs@Mel in the presence of ketamine (Figure 3, curve d) provide evidence for H-bond formation of ketamine with the surface of the AuNPs@Mel leading to NPs aggregation.

### 3.2. Optimization of reaction parameters

**Figure 4:**
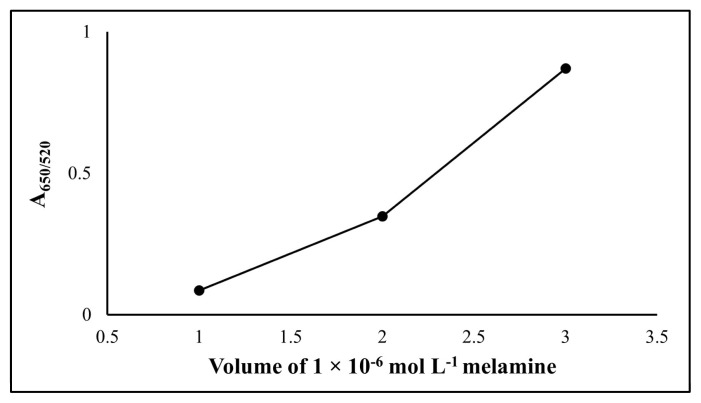
Effect of the different volumes of 1 × 10^−6^ mol L^−1^ melamine on corrected absorbance in the determination of 100 mg L^−1^ ketamine.

**Figure 5:**
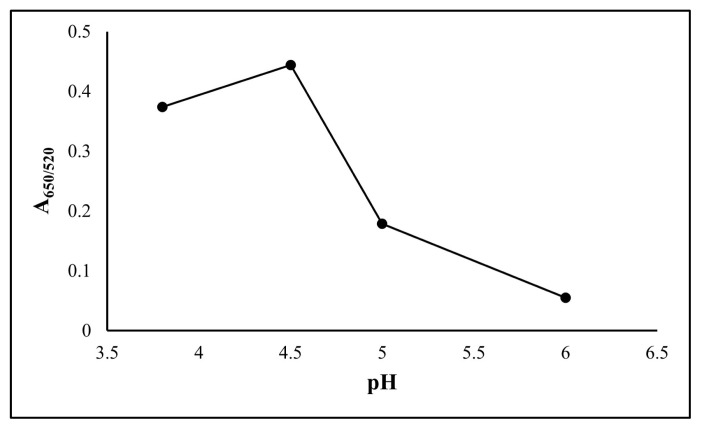
Effect of the working pH on corrected absorbance using 100 mg L^−1^ ketamine.

**Figure 6:**
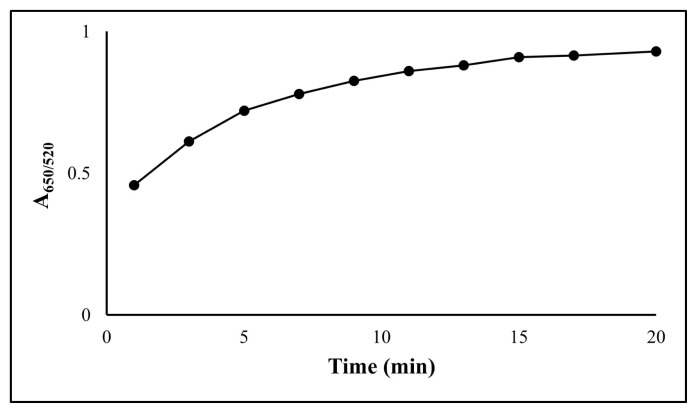
Effect of reaction time on the corrected absorbance using 100 mg L^−1^ ketamine.

The concentration and volume of melamine, pH, and reaction time were examined for method optimization. The appropriate values for the developed method are shown in Figures 4–6. In preliminary experiments with melamine concentrations of 1.0 × 10^−6^ and 1.0 × 10^−5^ mol L^−1^, the expected results were not obtained at 1.0 × 10^−6^ mol L^−1^, so the studies were performed with 1.0 × 10^−5^ mol L^−1^ of melamine. In subsequent volumetric experiments, 3 mL was determined to be the ideal amount for the modification of gold nanoparticles (Figure 4). At volumes higher than 3 mL, the AuNPs were aggregated. During pH optimization, experiments were performed at pH 3.8, 4.5, 5, and without adjusting pH (approximately 6), and it was observed that pH 4.5 was the best value (Figure 5). The acidity constant (as pK_a_) of ketamine is 7.5, and at the working pH of the analysed conditions, ketamine is more prone to form H bonds because it is in its acidic form. Thus, aggregation is observed more effectively. Finally, as no significant increase in absorbance was observed after the 15th min in the reaction time experiments, it was decided to make the measurements at the end of the 15th min (Figure 6).

### 3.3. Analytical figures of merit

**Figure 7:**
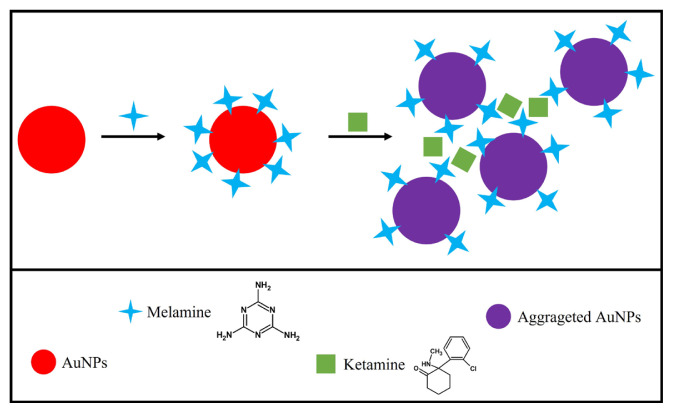
Aggregation of melamine-modified AuNPs (AuNPs@Mel) with ketamine.

Three hydrogen acceptor (NH) and six hydrogen donor (NH_2_) groups are present in each melamine molecule arising from the presence of exocyclic amino substituents and a triazine ring, respectively. As a result, melamine molecules can create nine hydrogen bonds at the maximum [41]. Ketamine, on the other hand, belongs to the category of cyclohexanones where one of the hydrogen atoms at position 2 is replaced by a 2-chlorophenyl group and the other by a methylamino group. Owing to the H-bonds formed between the relevant groups in the structures of ketamine and melamine (the latter functionalized on AuNPs), ketamine could be estimated within 10–100 mg L^−1^ levels with the developed method. Naturally, such interactions may not give rise to a colorimetric assay applicable in pure solution (i.e. without nanoparticles) unless the AuNPs@Mel are directed to aggregation. The representative scheme of the reactions is given in Figure 7.

**Figure 8:**
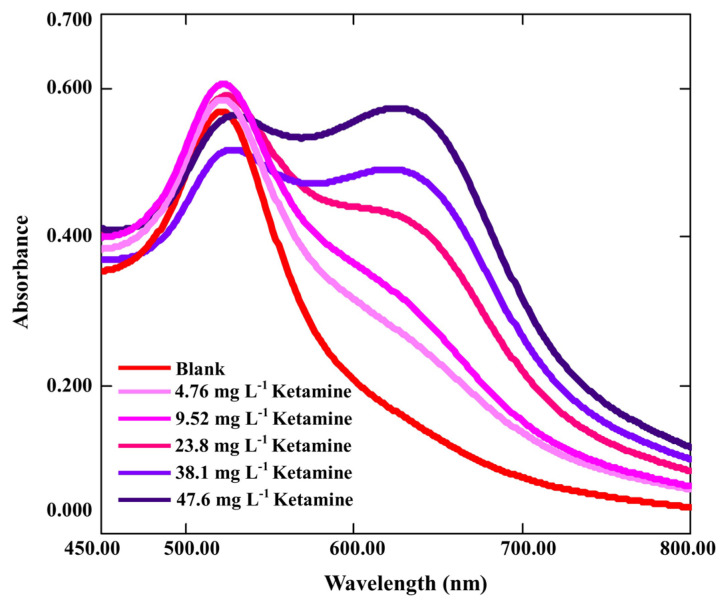
UV–vis spectra of AuNPs@Mel at different concentrations of ketamine.

When the proposed method was applied to different concentrations of ketamine, the obtained UV–vis spectra are shown in Figure 8. The calibration curve was drawn between ketamine concentrations and corrected absorbance values (Abs_(650/520)_). Ketamine solutions with final concentrations ranging from 4.76 to 47.6 mg L^−1^ yielded a calibration curve with good linearity under optimal conditions:


$${\text{Abs}}_{\left(650/520\right)}=1.6\times 10^{-2}\;{\text{C}}_{\text{Ketamine}}-8.32\times 10^{-2}\;(\text{r}=0.9981)$$

where the molar extinction coefficient for ketamine was ɛ = 4.43 × 10^3^ L mol^−1^ cm^−1^. For the developed method, LOD and LOQ were 1.5 mg L^−1^ and 5.0 mg L^−1^, respectively (LOD = 3σ_bl_/m and LOQ = 10σ_bl_/m, where σ_bl_ denotes the standard deviation of a blank and m is the slope of the calibration curve). For each concentration performed on three replicate analyses, and depending on the concentration, the RSD (relative standard deviation) of a particular set of results ranged from 5.2% to 8.2%. With the proposed method, the intra-assay and inter-assay measurements gave coefficients of variation (CVs) as 5.7% and 8.5%, respectively (N = 3).

In the light of these analytical results, superiority was obtained in terms of LOD value compared to that of the gold nanoparticle method in the literature which was 2.70 × 10^−5^ mol L^−1^ (it is equal to 6.4 mg L^−1^) [42]. Modification of gold nanoparticles with melamine to yield AuNPs@Mel allowed to increase the sensitivity.

### 3.4. Analysis in FBS

**Figure 9:**
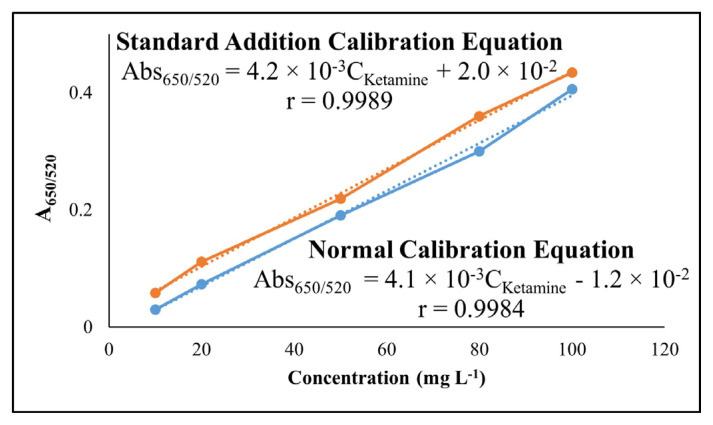
Unspiked and standard addition calibration curves for ketamine in FBS sample.

It is especially important that the developed method for ketamine analysis is applicable to real samples such as biological material. Therefore, fetal bovine serum was selected as the real sample, and samples were prepared as detailed in the “Materials and methods” section. According to the developed method, the calibration curve was created for ketamine estimation. Lastly, for the proposed method, the addition of ketamine standards to FBS was performed so as to construct the calibration curve at 10 mg L^−1^ concentration. Standard additions of ketamine to the FBS sample gave the analytical result as 9.65 mg L^−1^ in conformity with the added amount. The results confirmed the successful application of the method to FBS real sample, and the acceptable parallelism between the unspiked calibration lines and the standard addition calibration lines supported this argument (Figure 9), indicating the absence of a significant chemical deviation from Beer’s law of optical densities.

### 3.5. Evaluation of ions and camouflage materials

**Figure 10:**
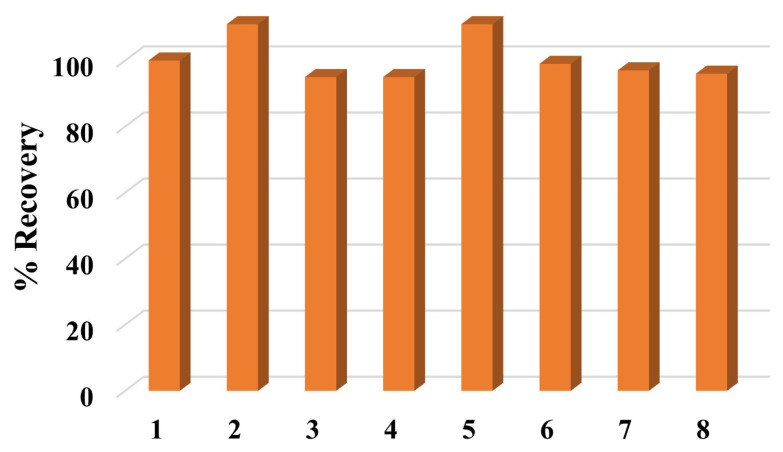
Recovery (%) of ketamine in the presence of ions and camouflage materials. (Ketamine (1), acetylsalicylic acid (2), paracetamol (3), K^+^ (4), Ca^2+^ (5), Cl^−^ (6), NO_3_^−^ (7), SO_4_^2−^ (8), Ketamine: 10 mg L^−1^ (initial concentration); interfering agents were used at the same concentration as ketamine.

To evaluate the interference effect of some ions and camouflage materials on the proposed method were also investigated. In Figure 10, we can see that ions did not have a significant effect on the developed method (95%–111%). In the same way, Figure 10 shows that acetylsalicylic acid (aspirin) and paracetamol-based painkiller-drug which can be used as camouflage materials during the transport of ketamine do not display any interference on the method in the presence of ketamine. Recoveries are 97% and 111% for acetylsalicylic acid and paracetamol, respectively.

### 3.6. Statistical comparison of the developed method with LC-MS/MS

For method validation, the literature LC-MS/MS method was performed to ketamine solutions [32]. The analyte solutions diluted in water to 1–30 μg L^−1^ concentrations were measured by LC-MS/MS and the following calibration equation was obtained:


$$\text{Peak Area}=8.6\times 10^{4}\;\text{C}+2.0\times 10^{4}\;(\text{r}=0.9988)$$

A solution at 50 mg L^−1^ concentration was used for the developed method and 5 μg L^−1^ concentration was used for LC-MS/MS method, and then, the calculated result was multiplied by the dilution factor. For N = 5 repeated analyses, the measurements were performed. No noticeable differences were observed between the accuracy and precision of the ketamine results obtained using the compared methodologies (Table). At 95% confidence levels for both tests, the Student t-test and F test were used to evaluate the population mean and variance statistically.

## 4. Conclusion

As the abuse of ketamine has shown an increasingly dangerous trend in recent years, it is of vital importance to develop rapid, simple, practical, and precise methods to determine ketamine and ketamine metabolites in complex samples. In this study, a quick, easy, and cheap colorimetric sensor for ketamine detection was developed depending on the H-bond interaction between ketamine and AuNPs@Mel. By using UV-vis spectrophotometry, it was possible to measure ketamine concentrations directly. Alternatively, the colour change of AuNPs@Mel from red to purplish-blue could be seen with the human eye. STEM, UV-vis spectrophotometry, and FTIR were used to characterize the synthesized and modified gold nanoparticles. In addition, the practical use of the proposed method was tested by performing the analyses in FBS as a real sample. Furthermore, the developed method has been successfully tested in some common ions and possible camouflage materials. This method was statistically validated by comparing its results with those of the prepublished LC-MS/MS method and agreement between results was confirmed. It is thought that the developed method will help the preliminary decision-making processes in criminal laboratories for ketamine analysis.

## Figures and Tables

**Table:** Comparing the proposed approach with the standard LC-MS/MS assay.

Method	Mean conc. (mg L^−1^)	SD (σ)	S^a^	t	t_table_	F	F_table_
Developed Method	46.59	4.59	-	-	-	-	-
LC-MS/MS Method	49.96	5.31	4.96	1.07	2.31	0.75	6.39

a In statistical calculations, the formulas were used as in the literature [43].
